# Limitations and optimizations of cellular lineages tracking

**DOI:** 10.1371/journal.pcbi.1012880

**Published:** 2025-04-14

**Authors:** Nava Leibovich, Sidhartha Goyal

**Affiliations:** 1 NRC-Fields Mathematical Sciences Collaboration Centre, National Research Council of Canada, Toronto, Ontario, Canada; 2 Department of Physics, University of Toronto, Toronto, Ontario, Canada; 3 Institute for Biomedical Engineering, University of Toronto, Toronto, Ontario, Canada; European Bioinformatics Institute, UNITED KINGDOM OF GREAT BRITAIN AND NORTHERN IRELAND

## Abstract

Tracking cellular lineages using genetic barcodes provides insights across biology and has become an important tool. However, barcoding strategies remain ad hoc. We show that elevating barcode insertion probability and thus increasing the average number of barcodes within the cells, adds to the number of traceable lineages but may decrease the accuracy of lineages inference due to reading errors. We establish the trade-off between accuracy in tracing lineages and the total number of traceable lineages, and find optimal experimental parameters under limited resources concerning the populations size of tracked cells and barcode pool complexity.

## Author summary

Many biological aspects can be examined using individual cellular lineages. For example, it allows us to investigate stem cell differentiation, cellular cooperation, stability of a phenotype, and more. To do so, the cells of interest are tagged with heritable identifiers called barcodes. One of the most common methods to label and track numerous lineages uses stochastic and combinatorial tagging. Here we investigate some properties of this random barcode labeling using a simple model, its mathematical analysis, and simulation. In particular, we examine the number of traceable lineages and the accuracy of lineages identification, while varying the initial barcode pool size, the labeling probability, and the barcode reading errors. We show a possible tradeoff between the accuracy of lineage identification and the number of tagged cells. Accordingly, careful planning of an experiment - corresponding to the required accuracy and needed number of tracked lineages - will be informed by our approach.

## Introduction

Cellular barcoding is a technique in which individual cells of interest are tagged with heritable identifiers called barcodes. Some barcoding techniques are based on unique insertion sites, but more commonly cells are labeled with unique nucleic acid sequences that can be tracked through space and time potentially providing insights into cellular behavior across a variety of biological contexts[1,2]. For example, it has been used to study lineages of T-cells examining migration patterns of families of antigen-specific CD8^+^ T cells [3], hematopoietic stem cells after transplantation [4,5], clonal dynamics of cancer cells to analyze metastatic progression [6–9], and mapping axonal projections for determining its diverse patterns [10] to name only a few. Tracking of cell lineages over time requires the insertion of a unique set of barcodes into each cell, propagating the cell population over time, and accurate reading of all barcode sets. Ideally, it allows us to detect clones and associate them with their lineages through time.

The insertion of barcodes into cells may be carried out by various procedures [11–14]. For small population of cells it has been done by manually assigning individual barcodes to cells one-by-one and thus guaranteeing a unique barcode for each cell [11,12]. Currently, the most common, robust, and efficient method to barcode a population of cells relies on the production of a large pool of barcoded vectors (e.g., viruses) which deliver the barcodes into the cells [13]. Importantly, by infecting an ensemble of cells with a pool of barcoded viruses, the actual number of viruses that will enter any given cell is a stochastic process: some cells may absorb more than one infectious agent while others may not absorb any [4,5,15]. Thus, this method, inherently, produces non-injective barcode-cell matching, meaning a given barcode can be inserted into more than one cell, and some cells may have more than one barcode. Yet, for a sufficient complexity of the barcode library, each cell’s set of labeling barcodes is presumably unique [16].

The barcoding procedure allows for the identification of lineages of many cells where the barcodes can be read using different approaches. Early approaches relied on bulk DNA sequencing which provides an accurate characterization of the clonal dynamics but provides no information on the cell state. Whereas the new single-cell sequencing methods that measures RNA levels have the ability to provide both lineages and cell state information [2,17]. Each method of sequencing; either the DNA or RNA sequencing, presents some advantages alongside some challenges concerning their preparation, amplification, extraction, reading, and data analyzing techniques [18–20]. In this paper we focus on the case of single-cell RNA sequencing which provides the expression profiles of individual cells but also presents an issue because some barcodes may be expressed at low levels, leading to reading errors called ‘dropouts’ where some barcodes may not be read in a cell [21–24]. Additionally, failure to detect barcode sequences when using transcriptomic data may be due to epigenetic silencing over time, especially with cell fate conversions [25].

**Fig 1:**
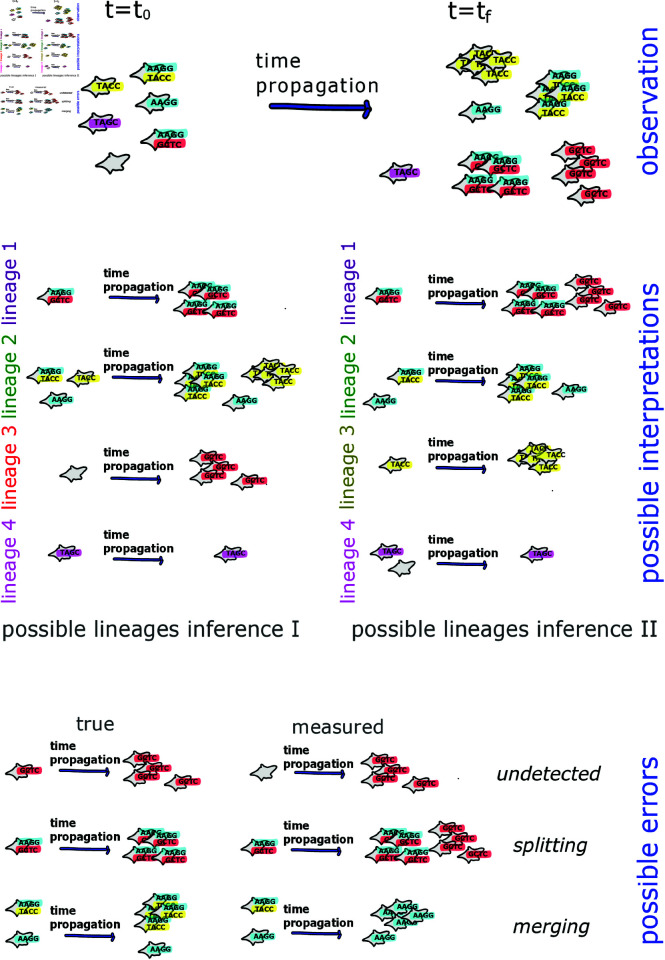
Single-cell barcoding allows tracking cell lineages with space and time. However, dropouts of barcodes throughout the observation complicate this task, even when the seeded barcode sets are unique (upper panel). The presence of dropouts gives rise to several lineage-structure interpretations that can be inferred from the measured barcoded cells. Here we illustrate only two possibilities of lineage inference, although other lineage inferences are possible as well (middle panel). Wrongly or unidentified lineages may occur due to unmeasured barcodes in one or more snapshots, associating two lineages as a single lineage, or identifying a single lineage as two separate ones (lower panel).

As mentioned, the randomness in the barcoding procedure may induce overlap between barcode sets across individual cells. This overlap along with the possibility of dropouts may lead to errors in identification of lineages. Note, that misidentifying clones due to dropouts may emerge even when every clone is infected by its unique set of barcodes. We illustrate this case in Fig 1 where one starts with some clones that were infected with unique barcode sets at some initial time. Then, after some propagation time where clones proliferate deferentially, the clones are re-observed. Here the aim is to associate clones measured at a later time with their ancestors to gain insight into the clonal dynamics. Note that some clones may have more than one barcode, and some clones might not have been infected at all. The figure shows how the stochastic barcode insertion and dropout can lead to errors in lineages identification and tracking. As shown in Fig 1, some observed clones at the later time, have not been measured at the earlier time hence some cells may be wrongly associated with erroneous lineages.

In the random barcode insertion method one of the tunable parameters is the multiplicity of infection (MOI), which is proportional to the ratio between the number vectors, for example of the number of viruses particles, to the total number of target cells [26,27]. Under the assumption that all vectors are equally infectious and all cells are equally susceptible to get infected, the number of barcodes inserted into each cell is well approximated by a Poisson distribution, and the average size of the barcode set s across all cells is taken to be the MOI. We note that deviations from Poisson assumption have been empirically reported [10,28,29], and we further discuss it below.

To overcome the lineage identification problem that emerges from barcode dropouts, the experiments are usually designed such that the MOI is sufficiently low, and the barcode library is highly diverse [15], as shown by the numbers from recent publications in Table 1. Low MOI aims to reduce the number of clones with more than one barcode and a highly complex barcode library decreases the probability of having the same barcode integrating into multiple cells. However, using lower MOI has a cost in terms of a larger fraction of unmarked cells and a higher diversity of barcode pool is expensive and may be excessive or unnecessary considering the desired accuracy of the empirical data, and the overall goal of the experiment [19]. Given the ubiquity of these techniques, understanding the cost-quality trade-off for more prudent experimental design will impact a wide research community.

**Table 1:** Cellular barcoding parameters in a variety of systems.

System [Ref.]	#cells (*S*)	#barcodes (*B*)	MOI (*M*)	% labeled cells
Embryonic data [15]	$7.4\times 10^{4}$	$∼10^{6}$	0 . 2 (used)	∼ 20*%*
			0 . 15 (measured)	
Hematopoietic stem cells [5]			1	∼ 85*%*
Clonal dynamics [28]	$2\times 10^{6}$	20 , 000	0 . 05 − − 0 . 1	5%–10%
Neuronal structure [10]	$∼10^{8}$ (theory)	$∼10^{18}$ (theory)	0 . 43 a	∼ 80*%*
	$∼10^{2}$ (exper.)	$∼10^{6}$ (exper.)		
Induced pluripotent stem cells [31]	170,000–230,000	50K–16,000 K	0.35–0.89	29.1%–59.1%
Patient-derived xenografts [32]			0 . 07 b	7 ± 4 . 99*%* c
Of breast cancer			0 . 13 b	12 . 18 ± 8 . 13*%* c
			0 . 23 b	20 . 35 ± 7 . 71*%* c
Clonal dynamics in reprogramming [29]		36 , 000	0.05–0.1	5%–10%
Cells fate [33]	5,000–10,000	$0.5\times 10^{6}$		
Synaptic networks [34]		$1.29\times 10^{6}$	∼ 0 . 15 d	∼ 8 . 57*%* e
			∼ 1 . 5 d	∼ 32 . 81*%* e
			∼ 15 d	∼ 44 . 44*%* e

^a^ Infected neurons contained 1 . 2 ± 0 . 1 barcodes each [10].

^b^ We estimated it using the assumption in [32] that it follows Poisson statistics.

^c^ Mean  ±  STD, see [32].

^d^ Unique barcodes mean  ±  STD for neurons/astrocytes: MOI  ∼ 0 . 15 : 1 . 07 ± 0 . 009 ∕ 1 . 03 ± 0 . 01. MOI  ∼ 1 . 5 : 1 . 42 ± 0 . 01 ∕ 1 . 14 ± 0 . 01. MOI  ∼ 15 : 1 . 6 ± 0 . 01 ∕ 1 . 2 ± 0 . 02, see [34].

^e^ We calculated it using the absolute number of infected and uninfected cells given in [34].

Here, we address this within a simple stochastic model for barcode integration, dropout, and cell population propagation through time. We find that there is an optimal range of MOI that maximizes the fraction of lineages tracked with high confidence, given the system’s properties. We note though, that we consider here the barcode sets’ overlap and dropout effects, while other error sources such as barcode swapping [30] are left for further research.

## Model

### Barcodes integration

We consider an ensemble of *S* cells at time *t* = 0. Then, we randomly “insert” barcodes from a pool with *B* unique barcode types into the cells. We note that *B* refers to the complexity of the barcode pool and is different from the total number of barcode carrying vectors.

The insertion procedure depends on both the susceptibility of cells and the barcode insertion probability. Assuming the infectedness of a vector is independent of the barcode type it is carrying—the insertion probability depends on the abundance of individual barcode types. For example, in a pool where all barcode types are equally abundant, the insertion probabilities are identical for all types. Depending on the MOI and barcode complexity, cells may range from having no barcodes to having a combination of several barcodes. Recall that due to the randomness of the insertion process, each barcode may be inserted into multiple cells, and hence the set of integrated barcodes for a cell is not guaranteed to be unique.

**Fig 2:**
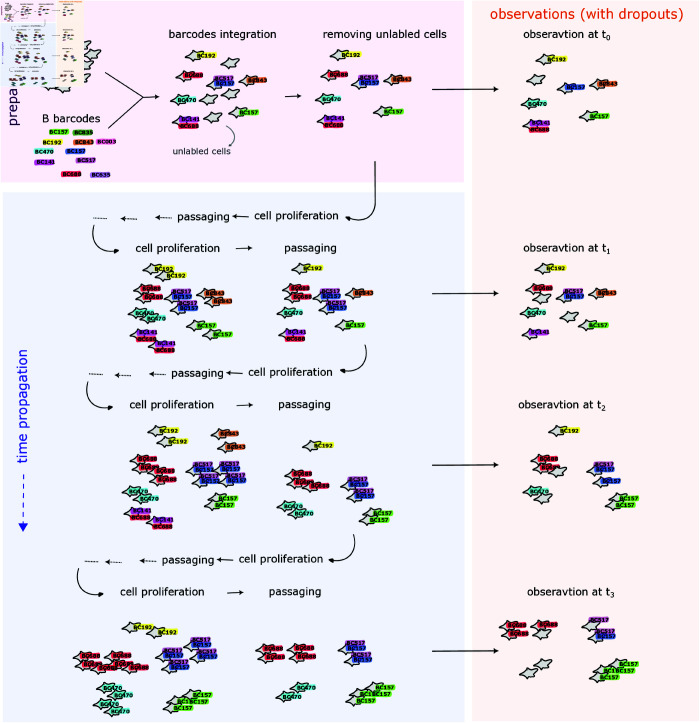
Scheme of the model: Begin with *S* prepared cells and a barcode pool containing *B* unique barcodes identified by nominal numbers. Barcodes integrate into cells based on the infection probability $p_{s,b,\text{In}}$. The labeled cells undergo multiple proliferation and passaging events over time. At specific times, the clone population is observed subject to barcode reading errors governed by the dropout probability of $p_{\text{Drop}}$.

**Fig 3:**
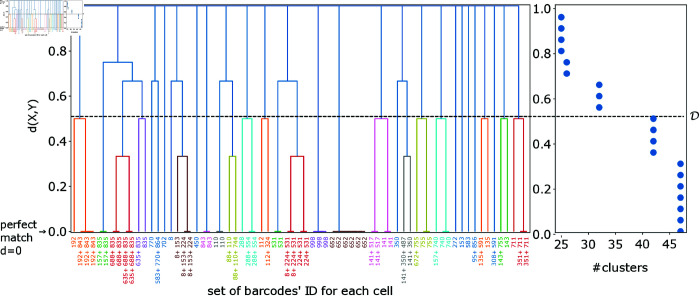
An illustration for the lineages identification procedure. Left panel: Each leaf represents a cell, characterized by its measured set of barcodes, denoted as $\left\{b_{\text{In}}\right\}$. Each individual barcode $b_{\text{In}}$ is identified and named by a unique integer number. A distance matrix *d* ( *X* , *Y* )  is computed for all cells to measure the dissimilarity between their barcode sets, see Eq. (6). This distance matrix is used to construct a dendrogram through agglomerative clustering. Right panel: We vary the threshold and count the number of inferred clusters. That value determines the required matching threshold for the lineages’ construction. Note that the data and threshold values shown in this illustration are for demonstration and visualization purposes only. The presented data set is truncated and small, whereas the actual systems examined in the manuscript involve thousands of cells.

Each element in the set *B*, is a unique barcode type noted as *b*, and each cell in the cell set is marked with *s*. The probability of each barcode type *b* to be inserted into a specific cell *s* is given by $p_{s,b,\text{In}}$, where ${\left(\right)}_{\text{In}}$ denotes “insertion.” Each cell is thus associated with an integrated set $B_{s,\text{In}}=\{b_{\text{In}}\}$ where $\left\{b_{\text{In}}\right\}$ are the barcodes types integrated into the cell *s* where $B_{s,\text{In}}\subseteq B$. Then, the probability of integrating exactly *L* different barcode types into the specific cell is given by


$$\begin{array}{ccc} P_{s}(L)=\underset{\text{of set}B}{\underset{L\text{-combinations}}{\sum }}\underset{b_{j}\in B\setminus \{b_{\text{In}}\}}{\underset{b_{i}\in \{b_{\text{In}}\}}{\prod \;}}p_{s,b_{i},\text{In}}(1-p_{s,b_{j},\text{In}}), & & \end{array}$$ (1)

where $L=|B_{\text{In}}|$ is the size (cardinal) of the set $B_{\text{In}}=\{b_{\text{In}}\}$, and  | *B* | = *B* is the barcode complexity. For simplicity we name each prepared barcode *b* ∈ *B* with a unique nominal number (see, for example, Figs 2 and 3). The summation over *L*-combinations of the set *B* refers to the summation over all subsets of *L* distinct barcodes from the set of barcodes *B*.

Although simple in construction, the above integration model is challenging to examine analytically. Therefore, we initially present mathematical results under the assumption that all barcode types have an equal probability of integration and that cells have uniform susceptibility, which leads to $p_{s,b,\text{In}}=p_{\text{In}}$ for every *b* and *s*. Afterward, we will discuss the implications of relaxing this assumption.

With this simplification, the probability of having a cell with a barcode set of size *L* is given by


P(L)=Binom(B,pIn)=BLpInL(1−pIn)B−L. (2)

In the limit of low insertion probability, i.e., $p_{\text{In}}\ll 1$, and high barcode complexity, *B* ≫ 1 as tabulated in Table 1, the size of the barcode set is approximately Poisson distributed [35],


$$\begin{array}{ccc} P(L)\approx \text{Poisson}(M)=\frac{e^{-M}M^{L}}{L!} & & \end{array}$$ (3)

where the mean number of barcodes per cell $\langle L\rangle =M\equiv p_{\text{In}}B$ is related to the MOI in Table 1. Here, the fraction of infected cells given by $1-e^{-M}$ is used to estimate *M*, and $p_{\text{In}}$ is determined by the barcode pool complexity *B* and the estimated *M*. Details about the binomial and Poisson distributions are discussed in pages 2-4 and Figs A-D in S1 Appendix, where we show simulation results in agreement with the analytical predictions. In addition, we show that the average number of total barcodes integrated across all cells is *M* ⋅ *S*, hence the MOI indeed is proportional to the ratio of the number of virus particles to the number of cells.

Deviations from the uniform integration case described above, where all cells are equally susceptible to being barcoded and all barcodes are equally infectious, have been reported in experimental scenarios [10,28,29]. These deviations present an over-dispersed distribution, where the variance of the measured distribution exceeds the mean value. To examine the case of biased integration, we simulated barcode integration where both barcodes integration frequencies and cells’ infection susceptibilities were not uniform, see pages 5–8 in S1 Appendix for details about the simulation. We compare the lineage tracking efficiency in both the uniform and biased cases below.

### Propagation

To pose the problem of lineage tracking, we propagated the barcoded populations of cells in time considering population growth and passaging events, where we keep the total population of cells fixed. The stochastic passaging results in the loss of many lineages, where briefly at each passaging event, the population of cells is randomly sampled and then it is allowed to grow back, where the passaging frequency and sampled cells percentage affect the number of surviving lineages. In pages 8–12 in the S1 Appendix we examined the effect of the frequency of passaging, and the sampling ratio on the number of surviving lineages. In the simulation presented, we propagated the system for a total of 15 generations but analyzed only the clone populations at generations 0, 5, 10, and 15. One expects that the number of lineages propagated through time may depend on the modeling details of the stochastic sampling and growth [36–39]. In pages 8-12 in S1 Appendix, we demonstrate that although this holds true, comparable outcomes were obtained with different assumptions, such as synchronized versus unsynchronized local rules, and implementations, such as Wright-Fisher versus Gillespie [36–39]. The results in the main text are presented with the simplest assumption of synchronized doubling-splitting of the population.

It is important to note that here we have considered neutral dynamics, which means all cells are equivalent such that no clone possesses an advantage over the others in their ability to propagate from one generation to the next one. This may not be generic as shown in multiple cases [28,29,40,41]. Given that selective clone dynamics are not correlated with barcode identity or barcode integration, it is not expected that the general aspects of the barcoding strategies discussed here will be significantly influenced by the specific nature of the dynamics.

### Dropouts

An important challenge in tracking lineages is barcode reading errors referred to as the “dropout” of a barcode—where one or more barcodes in a cell are not read. In general, the barcode reading quality improves with the sequencing depth and coverage. However, that comes at a higher cost. Therefore, the choice of sequencing depth and coverage is suggested to be an important aspect of the overall experimental design [42]. For RNA sequencing, reducing the dropout rate is challenging since it depends on the expression level and detection threshold. A high detection threshold may lead to false negatives or dropouts, where genes are mistakenly identified as not being expressed. Conversely, a low threshold may lead to false positives, meaning that genes are erroneously identified as being expressed due to measurement noise and thus affect the validity of reads [21–24]. Therefore here we assume the dropout rate is an outcome of the experiment and analyses pipeline and may not be a parameter that can be changed. The precise dropout percentage has been estimated by differential expression signature [22], where dropout percentages have been reported typically in the range of 10% to 50% [22,24,43–45].

We modeled the barcode dropouts by random elimination of some integrated barcodes from the tracked cells. The dropout probability is considered to be uniform i.e., it is independent of the integrated barcode set size, and the barcode or the cell identity [22]. However where measurements at multiple time points across changing cell states are made, the dropout rate may depend on other sources of noise such as the batch effects, and changes in the expected gene expression levels with cell types and states [22]. In this manuscript, we do not address these additional sources of noise, and focus on the conceptual role of the dropouts on lineage fidelity.

With a uniform dropout probability, $p_{\text{Drop}}$ the distribution of barcodes-set size is a binomial distribution where $p_{\text{In}}\to p_{\text{In}}(1-p_{\text{Drop}})$,


P(L)=Binom[B,pIn(1−pDrop)]=BL[pIn(1−pDrop)]L[1−pIn(1−pDrop)]B−L. (4)

Hence the *effective* MOI is now given by $\left\langle L\rangle =M=Bp_{\text{In}}(1-p_{\text{Drop}}\right)$. The number of cells with a given barcode also follows binomial distribution;


P(C)=Binom[S,pIn(1−pDrop)]=SC[pIn(1−pDrop)]C[1−pIn(1−pDrop)]S−C (5)

where *C* is the random variable that represents the number of cells with a given barcode, and *S* is the total number of cells prepared for integration. Here $\langle C\rangle =Sp_{\text{In}}(1\;-\;p_{\text{Drop}})=S\langle L\rangle ∕B=(S∕B)\;\cdot \;M$ is also linear with the MOI *M*. We emphasize that a partial reading of a barcode set due to dropout for a cell at any time is uncorrelated with reading errors at another time or other lineages. A comprehensive illustration, including the cell population and barcode library preparation, time propagation of tracked cells, and observations that are subjected to dropout probability are shown in Fig 2. The observed cells from all time points are then aggregated and analyzed for identifying lineages.

While considering only labeled cells, i.e., those with integrated barcodes, it is necessary to re-normalize the distribution in Eq. (4), results in $P(L_{\text{in labeled cells}})=P(L)∕$ $\left[1\;-\;{\left(1\;-\;p_{\text{In}}(1\;-\;p_{\text{Drop}})\right)}^{B}]=\text{ Binom}[B,p_{\text{In}}(1-p_{\text{Drop}})]∕[1-{\left(1-p_{\text{In}}(1\;-\;p_{\text{Drop}})\right)}^{B}\right]$. Therefore, the average number of barcodes within labeled cells is $\left\langle L_{\text{in labeled cells}}\rangle =B[p_{\text{In}}(1\;-\;p_{\text{Drop}})\right]$ $∕[1\;-\;{\left(1\;-\;p_{\text{In}}(1\;-\;p_{\text{Drop}})\right)}^{B}]$. We note that our analyses also take into account the probabilities of being labeled or unlabeled. This information becomes obscure when focusing solely on the barcoded cells. Therefore, throughout the paper, we will continue to include all cells, both labeled and unlabeled, in our analyses, as expressed in Eq. (4).

### Building lineages

The next step in our computational model is to construct the cellular lineages. Since the measurements across cells and time are uncorrelated from each other, we aggregated the barcode sets measured for all the cells throughout the propagation times together. To define lineages we performed clustering where the barcode sets are the clustering features. Due to the multiple barcode integration resulting in overlaps in barcode sets across cells, as well as the difficulty due to reading errors from the dropouts, the clustering procedure required choosing carefully both the similarity metric (‘distance’ between barcode sets) and the clustering threshold. First, we built a distance matrix between every cell pair using the dissimilarity ratio between their barcode sets.


$$\begin{array}{ccc} d(X,Y)\equiv 1-\frac{\left|X\cap Y\right|}{\left|X\cup Y\right|} & & \end{array}$$ (6)

where *X* , *Y* are the barcode sets, and  | . |  refers to their cardinals. Then, we performed hierarchical agglomerative (‘bottom-up’) clustering and matched together cells with their dissimilarities smaller than some threshold *D*. In Fig 3 we present an illustration of a dendrogram, where each terminal leaf represents a cell *s* with its own set of measured barcodes $B_{s,\text{In}}=\{b_{\text{In}}\}$, with each barcode $b_{\text{In}}$ being named with a unique nominal number. We examined three dissimilarity thresholds: one allowing any degree of overlap, one reducing the matching of clones by minimizing the dissimilarity threshold, and a third intermediate threshold that utilizes the known lineage count. The choice of the clustering threshold significantly impacts the results and thus requires careful selection, see results below and in pages 13–16 in S1 Appendix. The computational model, including code for generating the synthetic data and the analysis functions used to produce all plots, are provided in [46].

## Main results

Given the constraints imposed by barcode reading errors, primarily tied to the dropout probability, the main approaches employed to enhance the accuracy of lineage inference have been (1) to expand the barcode complexity, and (2) to reduce the MOI. Indeed, both approaches reduce the probability of any specific barcode to be integrated into multiple cells, and thereby limit the overlap between barcode sets across cells, but both come with challenges. Increasing the barcode complexity has a direct bearing on the cost—as both a bigger barcode size and a larger population of vectors are required. Additionally, larger libraries have a tendency for skewing and are more difficult and costly for sequencing since more reads are needed to maintain the sequencing depth required for signal-noise discrimination [19]. Yet, since the overlap probability approaches zero with increasing the barcode complexity, the marginal gain goes down with the barcode complexity. Having low MOI means a lower fraction of barcoded cells, hence a larger population is needed to have the same number of trackable lineages.

Consequently, given the constraints on resources and the permissible margin of error in lineage identification, expanding the barcode library or increasing the number of cells may not be essential for achieving the desired goal. In essence, a definite complexity in the barcode library with a specific range of MOI could suffice to meet observational requirements.

### Analytical examination of the uniform integration model

**Fig 4:**
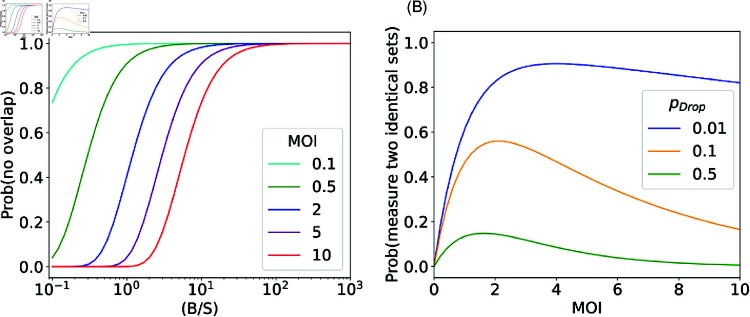
Analytic examination of the uniform integration model. (A) the probability of no overlapping between barcode sets follows Eq. (7). (B) the probability that two barcode sets are measured exactly the same after dropout events, following Eq. (9).

To develop an intuition, we first present an analytical examination of a model with uniform integration and dropout probabilities across all barcodes. The following analysis only addresses erroneous identification due to multiple integrations and dropouts, excluding lineages lost in propagation. As discussed above, errors in the lineages construction increase with the level of overlap between sets of integrated barcodes across cells. In this model, the probability of overlap between integrated barcodes across any two cells is directly proportional to the probability of multiple integration of a barcode into more than one cell. Using the Poisson approximation of Eq. 5 we obtained


$$P(\text{any overlap})\approx \overset{\infty }{\underset{C=2}{\sum }}\exp(-\langle C\rangle )\frac{{\left\langle c\right\rangle }^{C}}{C!}=1-\left(1+\frac{S}{B}M\right)\exp\left(-\frac{S}{B}M\right),$$

where *S* is the number of prepared cells, *B* is the barcode complexity, and *M* is the MOI. Note that the probability of no overlap is then


$$\begin{array}{ccc} P(\text{no overlap})=1-P(\text{any overlap})=\left(1+\frac{S}{B}M\right)\exp\left(-\frac{S}{B}M\right) & & \end{array}$$ (7)

which is exponentially decaying function with the variable $\left(\frac{S}{B}M\right)$. It means that for a given $\left(\frac{S}{B}\right)$ ratio, higher MOI leads to greater overlap between integrated barcode sets, see Fig 4A.

Next we examine the effect of the dropout probability on the barcode overlap. The probability that two cells with identical integrated barcode sets with size *L*, are also measured to have exactly the same set of barcodes after dropouts is


$$\begin{array}{ccc} \text{Prob}(\text{barcodes in cell 1}=\text{barcodes in cell 2}|L)={\left(2p_{\text{Drop}}^{2}-2p_{\text{Drop}}+1\right)}^{L}-p_{\text{Drop}}^{2L}, & & \end{array}$$ (8)

see derivation in pages 17-21 in S1 Appendix. Therefore, the probability to measured two identical sets across all possible *L*, is


$$\begin{array}{cccc} & & \text{Prob}(\text{measure two identically sets})=\\ & & \underset{L}{\sum }\text{Prob}(\text{barcodes in cell 1}=\text{barcodes in cell 2}|L)P(L)= & \\ & & \underset{L}{\sum }\left\{{\left[{\left(1-p_{\text{Drop}}\right)}^{2}+p_{\text{Drop}}^{2}\right]}^{L}-p_{\text{Drop}}^{2L}\right\}\frac{e^{-M}M^{L}}{L!}=e^{-2p_{\text{Drop}}[1-p_{\text{ Drop}}]M}-e^{-[1-p_{\text{Drop}}^{2}]M}. & \end{array}$$ (9)

As expected, the probability of measuring identical sets vanished in multiple cases: *M* = 0 since no cell is labeled, $p_{\text{Drop}}=1$ since all barcodes were dropped, and for any $p_{\text{Drop}}\neq 0$ when *M* → *∞*. For a general $p_{\text{Drop}}$ and *M* the above expression is a concave function with a maximum value at an intermediate value of MOI, see Fig 4B. These two analytical expressions presented in Eq. (7) and Eq. (9) provide the intuition for how the expected errors in lineage tracking and reconstruction depend on the three experimental parameters—population size *S*, barcode complexity *B* and the MOI *M*. We note that Eq. (7) captures the behavior when the lineages reconstruction approach is based on any non-zero overlap between barcode sets. A minimal overlapping threshold is described qualitatively by Eq. (9), since it is partially governed by measurements of identical (or nearly identical) sets, see additional discussion on this in pages 17–21 in S1 Appendix. Nevertheless, the above analysis provides the basic intuition for the interplay of different parameters. Below we present simulation results for the computational model with multiple passaging events of a large population of cells. Remarkably, the results of the full computation model showed the same qualitative behavior as our analysis presented in this section.

### Simulation results

#### Overlap threshold for lineages identification.

**Fig 5:**
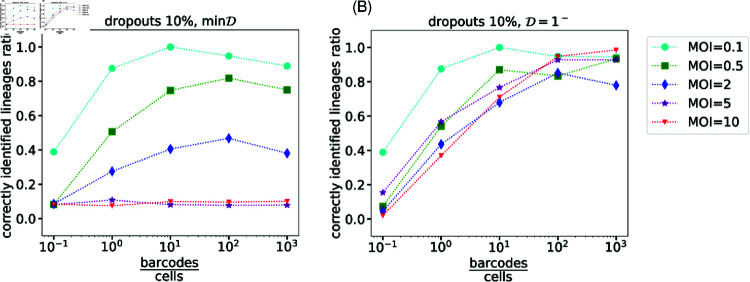
The dependence of barcode library complexity. We have examined the barcodes’ pool complexity using two lineages-construction approaches - min*D* and $D=1^{-}$ [panels (A) and (B) respectively]. Simulations suggest that increasing the diversity of potential seeded barcodes up to some complexity indeed implies improvements in the lineage tracking quality. Yet, saturation in the lineage tracking quality emerges beyond that required complexity, regardless of the lineages’ deduction strategies we examined. Here the number of cells is $S=10^{3}$ with 10% dropouts.

**Fig 6:**
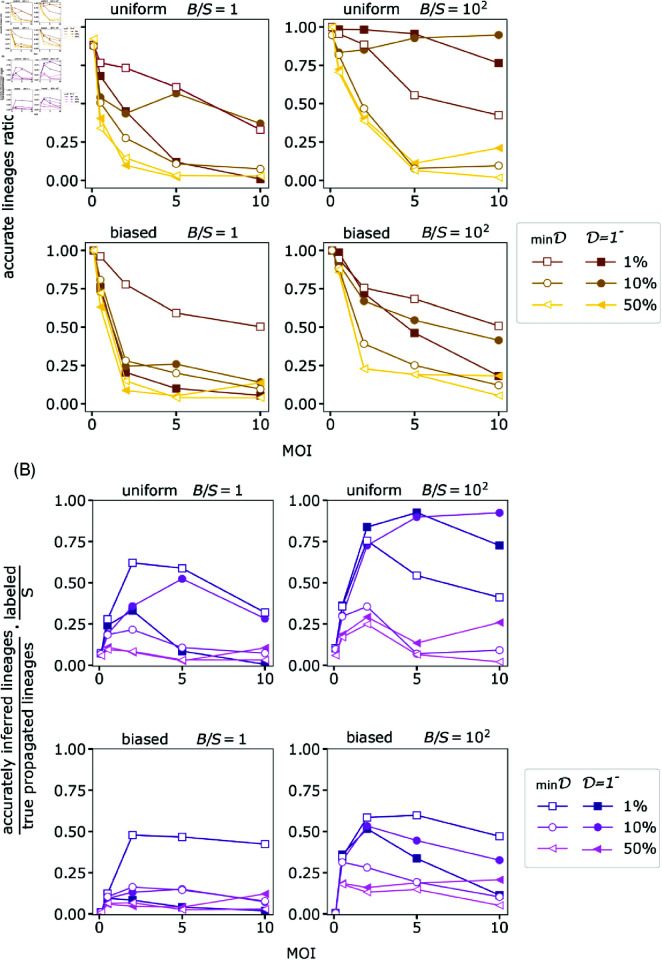
The dependence of MOI. (A) The accurately identified lineages ratio versus the MOI for various system features. The ratio is defined by the number of accurately inferred lineages over the number of true propagated lineages. (B) The percentage of accurately identified propagated lineages times the percent of labeled cells. For both panels, we present simulation results for diversities *B* ∕ *S* = 1 and $B∕S=10^{2}$ (left and right columns respectively), the uniform and biased integration (upper and lower rows correspondingly). We also examine the two lineages reconstruction strategies with a minimal dissimilarity matching *D* = min*D* (empty markers) and $D=1^{-}$ (full markers).

**Fig 7:**
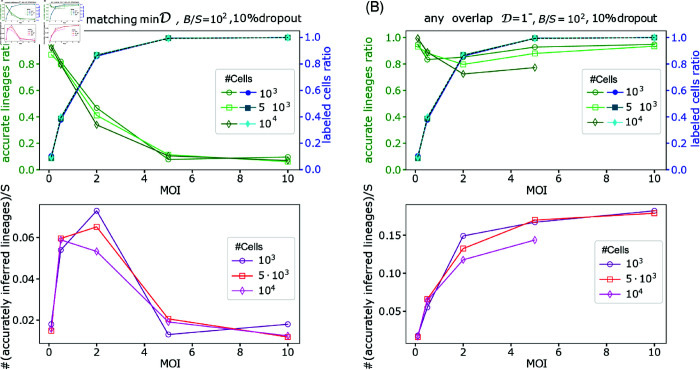
The dependence of MOI on the percentages of labeled cells and accurately identified lineages. Increasing the MOI results in increasing the number of infected cells (upper panels, blue shades, full markers), while the ratio of accurately identified lineages may decrease, depending on the system properties and the analysis strategy - minimal matching threshold *D* = min*D* and any non-zero overlap $D=1^{-}$ (green shades, open markers, shown in upper-left and upper-right panels correspondingly). Lower row: The ratio of lineages accurately observed. We show simulation results for *D* = min*D* (lower-left panel) and $D=1^{-}$ (lower-right panel). In here we use the number of cells as $S=10^{3},5\cdot 10^{3},10^{4}$, and the number of barcodes is 100-fold larger than the initial number of cells with 10% dropouts.

To build lineages, we aggregated the data from all cells throughout the propagation times and clustered their barcode sets. First, we built a distance matrix that captured the overlap ratio between the barcode sets for every pair of cells, see Eq. 6. Then, we performed hierarchical agglomerative (“bottom-up”) clustering and matched cells whose dissimilarity distance is smaller than a certain threshold *D* to be part of the same lineage, see illustration in Fig 3. We considered three lineages-building strategies. The first one identified two cells with a given lineage if their barcode set was observed with any non-zero overlap namely $D=1^{-}$, thus maximizing the matching of cells. In this approach, lineage identification errors emerged from multiple barcode integration. The other two thresholds we considered rely on the number of lineages they identified and thus depend on the system properties. For the threshold marked as *D* = * min* ⁡  *D*, we used the number of cells as a proxy for the maximum possible number of lineages. The last threshold we examined, $D=D^{*}$, is determined by the true number of lineages. We note that the choice of $D^{*}$ manifestly requires information that is a-priori not accessible. The lineages inferred using $D^{*}$ nearly perfectly recovered the true nature of the propagated lineages, see Fig. 1 in S1 Appendix. This provides support towards the applicability of our distance matrix *d* ( *X* , *Y* ) , i.e., it encapsulates the information required for lineage building. However, one remains with the challenge of choosing the appropriate threshold, without knowing the true number of lineages. We note that for low MOI, say  ≲ 0 . 5, there was almost no dependence on the choice of threshold since *d* ( *X* , *Y* )  is mostly binary, either 0 or 1. If the number of lineages cannot be inferred independently, rendering $D^{*}$ indeterminate, we were left with the two other thresholds. As shown in Figs 5–7, the threshold $D=1^{-}$, which maximizes cell matching, yielded good results at high MOI and with sufficiently highly diverse barcode pools. For low MOI, and especially for low complexity (*B* ∕ *S*) and biased barcode integration, we found that min*D* yields better (or at least not worse) results. See further discussion, including a comparison between the thresholds, and the variable threshold used in simulations, in pages 13–16 in S1 Appendix.

#### Barcodes library complexity.

We found that beyond a certain value, increasing the complexity of the barcode library did not necessarily result in a significant improvement in lineage inference quality. Nonetheless, a minimal barcode library complexity is needed to minimize the barcode overlap across cells. Fig 5 shows the dependence of the fraction of correct lineages as a function of *B* ∕ *S*. As expected, the fraction increases with complexity but importantly it saturates beyond a certain value of complexity, which increases with the MOI. Here, with a dropout rate of 10%, saturation emerges in value ranges between *B* ∕ *S* ∼ 10 − 100. That is in agreement with the empirical rule given in [19]. Notably, it is consistent with our analytical examination of the uniform integration model - there, the requirement that Prob(any overlap) is less than 1 ∕ *S* yields $S^{3∕2}\text{MOI}≲B$, as shown in Figs 4A and 5B where saturation emerges at $B∕S∼M\sqrt{S}$, see derivation in pages 22-23 in S1 Appendix.

Beyond the dependence on the complexity, Fig 5A highlights the strong dependence on the choice of the threshold. In particular, for *D* = min*D*, increasing barcode complexity did not improve lineage identification at large MOI. Conversely, for $D=1^{-}$ lineage identification improved for all of the examined MOI values as shown in Fig 5B. It signifies the remarkable advantage of using $D=1^{-}$ in large complexity scenarios where barcode overlap is improbable.

#### MOI range.

The analytical examination shown in Fig 4B highlights the potential non-monotonicity in the dependence on the MOI, where an intermediate value of MOI may be optimal. The intuition for that is as follows. On the one hand, at low MOI––which corresponds to very low barcode insertion probability—a larger fraction of cells is left unlabeled leading to fewer traceable lineages. On the other hand, for high MOI—which leads to multiple barcodes in individual cells—thus, misidentifying lineages due to dropouts becomes more probable.

To assess carefully the dependence of the MOI we analyzed four asymptotic possibilities arising from a combination of low and high barcode complexity and uniform and biased integration. We also consider the two lineage construction strategies - $D=1^{-}$ and *D* = min*D*.

Low MOI yielded good lineage identification—regardless of the dropout rates and the overlap threshold used for lineage reconstruction—see simulation results in Fig 6A. This is consistent with a low probability of overlap between the integrated sets of barcodes at low MOI as is also shown in Eq. (7) and Fig 4.

While lineage accuracy decreases with an increasing MOI, the number of tagged cells rises, suggesting a potential trade-off leading to maximizing the traceable number of lineages at an intermediate MOI value. Consistent with that intuition, the ratio of accurately observed lineages to the total initial number of cells showed a non-monotonous behavior where an intermediate range of MOI maximizes the number of accurately observed lineages, see Fig 6B. As a result, there exists an MOI range that is considered ‘optimal’ within the constraints of available resources. The latter refers to the practical limitations of the size of the initial cell ensemble *S*, and the library complexity *B*. Hence, to accurately track a certain number of lineages in scenarios where a slight decrease in lineage inference accuracy may be acceptable for the research objectives, it might be more feasible to increase the MOI from 0.1 to 0.5, rather than to augment the number of cells *S*, as is shown in Figs 6 and 7. Note that Fig 7 demonstrates the data collapse when scaled with *S*—i.e., the number of accurately identified lineages shows linearity with *S*.

In addition, we comment that the “optimality” of MOI may differ with the needed accuracy and even may depend on the choice of the accuracy index. For instance, one may choose MOI considering the correctly clustered *cells* instead of the correctly identified *lineages*. In pages 24–26 in S1 Appendix we compare the two cell clustering scores: the Fowlkes–Mallows (FM) index and the v-measure, with the ratio of accurately identified lineages for the same data. We observe that the cell clustering scores for two scenarios—MOI=0.5 with 50% dropouts and MOI=2 with 10% dropouts—are similar. However, when analyzed using the number of accurately identified lineages, there is a significant difference between the two scenarios. This highlights the importance of the choice of the accuracy criterion.

Hitherto, we have thoroughly discussed the trade-off in selecting the MOI, particularly evident in “less-than-ideal” systems characterized by low diversity, biased integration, or inadequate clustering threshold. Importantly, we find nonetheless that for an “ideal” system—with a sufficiently diverse barcode pool, uniform barcode integration, and employing the non-zero overlap threshold for lineage construction—increasing the MOI does not necessarily compromise the quality of lineage identification. Consequently, within the examined range, a significant trade-off in MOI selection is not apparent in an “ideal” setup, as illustrated in Figs 6 and 7.

Quantitatively, the optimal MOI for maximizing the number of accurately identified lineages may vary depending on properties of the underlying experiment, such as the barcode pool skewness, the cell susceptibility, the homogeneity of cell growth, the passaging frequency, the percentage of the sampled subculture, and the dropout rate. Some experimental objectives may allow fewer tracked lineages for greater accuracy, thus the requirements and goals of the experimental study must be considered. Evident from our work is that careful selection of the MOI is crucial due to its significant impact on the overall outcomes, including the number of tracked lineages and their accuracy. Practically, the MOI can be determined through simulations or by using a small-scale experimental “sandbox” system.

#### Deviation from uniform barcodes integration.

As mentioned, in the uniform integration of barcodes, we assumed that the barcodes have equal chances to get inserted into a cell. In addition, we considered all cells to be equally susceptible of being barcoded. Nevertheless, in real experimental scenarios that might not be the case [10,28,29]. Still, as briefly mentioned, the above statements are valid even for non-uniform distributed barcodes, where certain barcodes are more probable to be delivered to cells. We examine such a scenario; where both cell susceptibility to be infected and barcode library are non-uniform. A qualitative similar behavior is obtained; the number of correctly inferred lineages presents non-monotonous behavior peaking at intermediate MOI, see simulation results in Fig 6.

## Discussion

Careful planning is needed for tracking lineages using genetic barcoding. Here we analyzed the impact of many key experimental parameters—the mulitplcity of infection (MOI), barcode pool complexity, reading errors leading to barcode dropout, and biases in barcode abundance and integration rates—on the number of traceable lineages. We used a stochastic model that along with above parameters incorporated cell passaging.

Our main finding is that in most conditions, there is an optimal MOI that maximizes the number of accurately tracked lineages. We show that this comes from a trade-off—increased number of labeled lineages as MOI increases, but at high MOI the chances of having the same barcode in multiple cells can damage the quality of lineage identification. We note though that in specific scenarios—with a sufficient barcode complexity, uniform barcode integration, and an appropriate lineages-construction approach such as using $D=1^{-}$ or ∗* —we find that increasing MOI may not necessarily damage the quality of lineage identification.

An important cost consideration in lineage tracking experiments is the barcode complexity. Our findings indicate that while a minimal barcode complexity is necessary, there is only a marginal benefit in exceeding a certain complexity threshold, in agreement with the model presented in [2]. Furthermore, we show that it is crucial to examine and validate the quality of the barcodes integration step—the diversity and the uniformity of integrated barcodes—to determine the parameters (threshold *D*) for accurate lineages reconstruction.

Overall, we show that the various experimental parameters combine in non-trivial ways to impact the number of traceable lineages. Yet, an intuition about them can be derived by simulations similar to those presented here and if possible in parallel by using a small-scale experimental “sandbox” system. As an example, we lay out a possible experiment. Let us assume that the aim is to track at least 100 lineages. Fig 7 shows that one may choose to prepare 10^4^ cells with MOI = 0.1 and track 100 lineages with up to 10% tracking error. However, if we can compromise on accuracy, say up to 30% tracking error, we can reduce the cells cost by half and start with 5 ⋅ 10^3^ cells with MOI = 0.5. This illustrates the contextual nature of the choice of these various parameters we have analyzed here, and hope this work provides valuable insights for experiment planning in this rapidly expanding field.

## Supporting information

S1 Appendix Appendices. (PDF)
